# Prevalence, predictors and outcome of hypofibrinogenaemia in trauma: a multicentre observational study

**DOI:** 10.1186/cc13798

**Published:** 2014-03-26

**Authors:** Jostein S Hagemo, Simon Stanworth, Nicole P Juffermans, Karim Brohi, Mitchell Jay Cohen, Pär I Johansson, Jo Røislien, Torsten Eken, Paal A Næss, Christine Gaarder

**Affiliations:** 1 Department of Research, Norwegian Air Ambulance, Stiftelsen Norsk Luftambulanse Postboks 39 1441, Drøbak, Norway; 2 Department of Anaesthesiology, Oslo University Hospital, Avdeling for Anestesiologi/Ullevål Postboks 4950 Nydalen 0424, Oslo, Norway; 3 National Health Service Blood & Transplant and Oxford Radcliffe Hospitals, John Radcliffe Hospital, Headley Way, Headington, OX3 9DU, Oxford, Headington, UK; 4 Department of Intensive Care Medicine, Academic Medical Centre, Postbus 22660 1100 DD, Amsterdam, the Netherlands; 5 Laboratory of Experimental Intensive Care and Anesthesiology, Academic Medical Centre, Postbus 22660 1100 DD, Amsterdam, the Netherlands; 6 Trauma Sciences, Blizard Institute, Barts and the London School of Medicine and Dentistry, Queen Mary University of London, The Blizard Building 4 Newark Street E1 2AT, London, UK; 7 Department of Surgery, San Francisco General Hospital, University of California San Francisco, 1001 Potrero Avenue CA 94110, San Francisco, CA, USA; 8 Section for Transfusion Medicine, Capital Region Blood Bank, Rigshospitalet, University of Copenhagen, Klinisk Immunologisk Afdeling Rigshospitalet Blegdamsvej 9 2100 København Ø, Copenhagen, Denmark; 9 Department of Surgery, University of Texas Medical School, Houston 6414 Fannin Street 77030 TX, USA; 10 Department of Biostatistics, Institute of Basic Medical Sciences, University of Oslo, Postboks 1122 Blindern 0317, Oslo, Norway; 11 Department of Research and Development, Division of Emergencies and Critical Care, The Oslo University Hospital Trauma Registry, Oslo University Hospital, Traumeregisteret Avdeling for Anestesiologi/Ullevål Postboks 4950 Nydalen 0424, Oslo, Norway; 12 Department of Traumatology, Oslo University Hospital, Avdeling for Traumatologi Postboks 4950 Nydalen 0424, Oslo, Norway

## Abstract

**Introduction:**

Exsanguination due to trauma-induced coagulopathy is a continuing challenge in emergency trauma care. Fibrinogen is a crucial factor for haemostatic competence, and may be the factor that reaches critically low levels first. Early fibrinogen substitution is advocated by a number of authors. Little evidence exists regarding the indications for fibrinogen supplementation in the acute phase. This study aims to estimate the prevalence of hypofibrinogenaemia in a multi-center trauma population, and to explore how initial fibrinogen concentration relates to outcome. Also, factors contributing to low fibrinogen levels are identified.

**Methods:**

Patients arriving in hospital less than 180 minutes post-injury requiring full trauma team activation in four different centers were included in the study. Time from injury, patient demographics, injury severity scores (ISS) and 28 days outcome status were recorded. Initial blood samples for coagulation and blood gas were analyzed. Generalized additive regression, piecewise linear regression, and multiple linear regression models were used for data analyses.

**Results:**

Out of 1,133 patients we identified a fibrinogen concentration ≤1.5g/L in 8.2%, and <2 g/L in 19.2%. A non-linear relationship between fibrinogen concentration and mortality was detected in the generalized additive and piecewise linear regression models. In the piecewise linear regression model we identified a breakpoint for optimal fibrinogen concentration at 2.29 g/L (95% confidence interval (CI): 1.93 to 2.64). Below this value the odds of death by 28 days was reduced by a factor of 0.08 (95% CI: 0.03 to 0.20) for every unit increase in fibrinogen concentration. Low age, male gender, lengthened time from injury, low base excess and high ISS were unique contributors to low fibrinogen concentrations on arrival.

**Conclusions:**

Hypofibrinogenaemia is common in trauma and strongly associated with poor outcome. Below an estimated critical fibrinogen concentration value of 2.29 g/L a dramatic increase in mortality was detected. This finding indicates that the negative impact of low fibrinogen concentrations may have been previously underestimated. A number of clinically identifiable factors are associated with hypofibrinogenaemia. They should be considered in the management of massively bleeding patients. Interventional trials with fibrinogen substitution in high-risk patients need to be undertaken.

## Introduction

Despite the implementation of damage control resuscitation principles, exsanguination remains a frequent cause of death in hospital [1,2]. Early coagulopathy is identified in 10 to 34% of patients arriving in hospital and is associated with increased mortality [3]. Fibrinogen plays a pivotal role in coagulation as it is converted into fibrin, which in conjunction with platelets forms a stable blood clot as the end haemostatic product [4]. Low fibrinogen levels or inefficient fibrinogen utilisation may adversely impact on patient outcomes.

Besides low fibrinogen levels due to blood loss and increased consumption, a number of factors affect fibrinogen metabolism in a massively bleeding trauma patient. Hypothermia reduces fibrinogen synthesis [5] whereas acidaemia following hypoperfusion leads to increased breakdown of fibrinogen [6]. Dilution of fibrinogen by crystalloid fluids and reduced fibrin inter-linkage by synthetic colloids has been demonstrated [7]. In addition to reduced fibrinogen availability, there are indications that fibrinogen utilisation is reduced in patients with trauma coagulopathy. This finding is associated with increased levels of soluble thrombomodulin and increased protein C activation [8,9].

The role of fibrinogen in massively bleeding trauma patients has been subject to increasing interest over the past few years. However, little evidence exists regarding the incidence of hypofibrinogenaemia and the factors that contribute to it. Several authors suggest a possible benefit from an earlier and more aggressive fibrinogen substitution strategy [7,10-13]. Current European guidelines recommend fibrinogen substitution at fibrinogen concentrations less than 1.5 to 2.0 g/l during uncontrolled bleeding [14]. Fibrinogen supplementation can be achieved by administering cryoprecipitate, fresh frozen plasma or fibrinogen concentrate, but levels of evidence to indicate the optimal source or schedule for fibrinogen supplementation in trauma are poor [10]. Measurement of fibrinogen concentration by conventional methods is time consuming and may delay necessary fibrinogen administration. Point-of-care assessments of haemostasis such as thrombelastography and thrombelastometry may provide more rapid indicators of the need for treatment [15].

The purpose of this multicentred international study was to estimate the prevalence of hypofibrinogenaemia in the initial phase after traumatic injury and to explore how the initial fibrinogen concentration relates to mortality in trauma patients. We also aimed to identify factors contributing to low fibrinogen levels.

## Materials and methods

### Patient selection and data collection

Data were collected from trauma patients admitted over a 2-year period from 1 March 2008 to 28 February 2010 in four different trauma centres (San Francisco General Hospital, USA; Royal London Hospital, UK; Radcliffe Hospital, UK; Oslo University Hospital, Norway). Criteria for inclusion were patients 18 years or older initiating full trauma team response in accordance with internationally accepted criteria for major trauma centres. Patients were excluded if the time from injury to arrival exceeded 180 minutes or was not known. All data were collected prospectively as part of an observational study, except for Oslo where data were retrieved from The Oslo University Hospital Trauma Registry. The study was performed in accordance with ethical regulations in the respective centres (see Acknowledgements). All patients included prospectively gave their consent to participate in the study. For patients included retrospectively, an exemption from informed consent was approved by the local Privacy and Data Protection Officer. Data collected included: age, gender, mechanism of injury, Injury Severity Score (ISS), the maximum Abbreviated Injury Scale (AIS) score per ISS body region, time from injury and 28-day survival. Along with fibrinogen concentration, the International Normalized Ratio (INR) and platelet count are known predictors of massive transfusion and mortality [16,17], and were therefore also included in the analyses. Base excess (BE) was collected as a surrogate marker of hypoperfusion, which in the trauma setting is highly correlated to blood loss [18].

### Sampling methods

Arterial blood samples for fibrinogen concentration, INR and platelet count were collected in citrated tubes. Heparinised syringes were used for BE sampling. All samples were collected shortly after arrival in the trauma room and were analysed without delay. Fibrinogen concentration was analysed by the Clauss method, and a value ≤1.5 g/l was defined as hypofibrinogenaemia. Prothrombin time was converted to the INR in accordance with the analytical system of each respective laboratory. BE was determined by conventional blood gas analysers. In this study we defined patients with BE less than –5.0 mEq/l as hypoperfused.

### Statistical methods

Summary data are presented as number (percentage) and mean (standard deviation) or median (quartiles) where appropriate. The AIS and ISS were treated as continuous variables. Student’s *t* test was applied for comparison of fibrinogen concentrations in groups based on outcome.

To explore the initial fibrinogen concentration as a predictor of 28-day mortality, we fitted a multiple logistic regression model, adjusting for the following covariates: ISS, age, time from injury, BE, INR, platelet count, gender and mechanism of injury.

Several of the independent variables, among them the initial fibrinogen concentration, were suspected to have a nonlinear relationship with the dependent variable [19]. In addition to a standard multiple logistic regression model, we thus also fitted a piecewise linear regression model [20] and a generalised additive regression model (GAM) [21]. These three regression models represent increasing levels of flexibility in the functional relationship between the dependent and independent variables. The standard logistic regression model assumes a linear relationship between the independent variable and the dependent variable over the whole range of the observed values for the independent variables. Piecewise linear models, on the other hand, refine this approach by assuming a series of linear segments and accompanying breakpoints. Finally, the GAM is a generalisation of generalised linear models and imposes minimal restrictions on the underlying relationship between the independent variable and the dependent variable by fitting a suitable spline function; that is, a set of polynomials. The optimal spline was chosen using cross-validation [21]. We used Akaike’s Information Criterion to compare the different regression models [22].

To identify possible predictors of low fibrinogen concentration we fitted two multiple linear regression models with fibrinogen concentration as the dependent variable. In the first model, BE, time from injury, age, gender and ISS were entered as independent variables. In the second model, ISS was replaced by separate AIS scores for the five different ISS body regions: head and neck; face; chest; abdomen and pelvic contents; and extremities and pelvic girdle.

All statistical analyses were performed using R 2.15/3.0.0 (R Core Team, 2013; R Foundation for Statistical Computing, Vienna, Austria). Results from the three multiple logistic regression models are presented as the odds ratio (OR) (95% confidence interval). For the linear multiple regressions, results are presented as standardised β coefficients (95% confidence interval). *P* < 0.05 was considered statistically significant.

## Results

A total of 1,133 patients from the four centres were included. Descriptive data for the study population are presented in Table 1. Hypofibrinogenaemia (≤1.5 g/l) was identified in 93 (8.2%) of the patients. Fibrinogen concentration less than 2.0 g/l was found in 211 (19.2%) patients. The mean (standard deviation) fibrinogen concentration on arrival was 2.68 (0.75) g/l for 28-day survivors and 1.95 (1.21) g/l for nonsurvivors (*P* < 0.001).

**Table 1:** Descriptive data for the study population

	**Total**	**Base excess less than −5**
Number of patients	1,133	177
Age (years)^a^	37.2 (24.7, 49.7)	37.0 (24.5, 49.5)
Gender (% male)	76.3	76.8
Mechanism of injury (% blunt)	88.3	78.5
Injury Severity Score	16.1 (14.2)	28.9 (16.0)
Base excess (mEq/l)	–2.48 (4.29)	−9.82 (4.6)
International Normalized Ratio	1.11 (0.24)	1.19 (0.22)
Platelet count (10^9^/l)	233.3 (64.4)	225.4 (77.0)
Fibrinogen (g/l)	2.62 (0.83)	2.05 (0.94)
Time from injury (minutes)	65.4 (37.7)	70.1 (38.8)
Survival (%)	91.3	72.7

Values presented as mean (standard deviation). ^a^Presented as median (quartiles).

**Figure 1:**
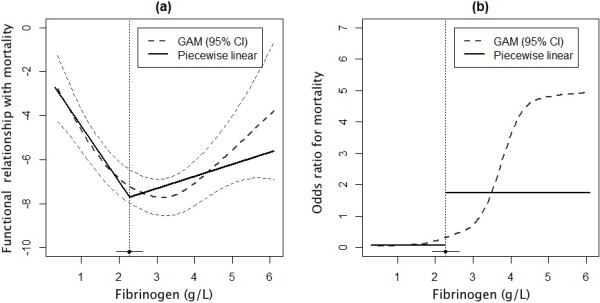
**Multivariable generalised additive model and piecewise linear model for relationship between fibrinogen concentration and 28-day survival.** Results from the multivariable generalised additive model (GAM) and the piecewise linear model for the relationship between fibrinogen concentration and 28-day survival, adjusted for Injury Severity Score, age, time from injury, mechanism of injury, base excess, International Normalized Ratio, platelet count and gender. The functional relationship is clearly nonlinear **(a)**, resulting in a corresponding nonconstant odds ratio across the observed range of fibrinogen values **(b)**. For the piecewise linear model, the breakpoint (95% confidence interval (CI)) is estimated at 2.29 (1.93, 2.64).

The fitted functional association between fibrinogen concentration and mortality rates using the GAM and the piecewise linear regression model is depicted in Figure 1a. We observe that a traditional linear logistic regression model is inadequate, and that a model allowing for a change in OR across the observed range of fibrinogen concentrations is needed. Measured by Akaike’s Information Criterion, the GAM is the best of the fitted models. However, a piecewise linear regression model also has strong support in the data. A traditional logistic regression model has no support in the data. Figure 1b shows the corresponding OR for the GAM and for the piecewise linear model. Compared with the GAM, the piecewise linear logistic regression model is more intuitively interpreted for clinical purposes, and is summarised in Table 2 along with results from the traditional linear logistic regression model for comparison.

**Table 2:** Linear and piecewise linear multiple logistic regression models with 28-day mortality as the dependent variable

	**Linear model**		**Piecewise linear model**		
	**Odds ratio v**	***P *** **value**	**Segment**	**Odds ratio (95% CI)**	***P *** **value**
Fibrinogen (g/l)^a^	0.46	< 0.001	Lower	0.08	< 0.001
				(0.03, 0.20)	
	(0.31, 0.67)				
			Upper	1.77	0.076
				(0.94, 3.32)	
Injury severity score^b^	1.03	0.008	Lower	1.18	< 0.001
				(1.10, 1.27)	
	(1.01, 1.05)				
			Upper	0.93	0.001
				(0.89, 0.97)	
Age (years)	1.05	< 0.001		1.04	< 0.001
	(1.03, 1.06)			(1.02, 1.06)	
Time from injury (minutes)	0.99	0.166		0.99	0.018
	(0.99, 1.00)			(0.98, 1.00)	
Mechanism of injury (penetrating)	0.73	0.546		0.33	0.06
	(0.25, 1.90)			(0.10, 1.05)	
Base excess (mEq/l)	0.90	< 0.001		0.92	0.002
	(0.85, 0.95)			(0.87, 0.97)	
International normalized ratio	3.21	0.012		1.65	0.29
	(1.33, 8.53)			(0.65, 4.18)	
Platelet count (10^9^/l)	1.00	0.61		1.00	0.92
	(1.00, 1.00)			(1.00, 1.00)	
Gender (male)	0.45	0.006		0.33	0.001
	(0.26, 0.81)			(0.18, 0.62)	

^a^Breakpoint for fibrinogen is 2.29 g/l (95% confidence interval (CI): 1.93, 2.64). ^b^Breakpoint for Injury Severity Score is 25.7 (95% CI: 21.8, 29.7).

A statistically significant breakpoint for the association between fibrinogen concentration and mortality was found at a fibrinogen concentration of 2.29 g/l (1.93, 2.64). In the segment below this breakpoint, the OR for mortality was 0.08 (0.03, 0.20); that is, a 90% reduction of the odds of mortality for each unit increase in fibrinogen concentration. In the segment above the breakpoint, the relationship was not significantly different from zero.

A nonlinear relationship with mortality was found also for ISS in our data. A breakpoint was identified for ISS at 25.7 (21.8, 29.7). ORs for mortality were 1.18 (1.10,1.27) for the lower segment and 0.93 (0.89, 0.97) for the upper segment.

In the multiple linear regression model with fibrinogen concentration as the dependent variable, the BE, time from injury, age, gender, and ISS were all statistically significant independent variables (Table 3, ISS model). In the second model, where the ISS was replaced by the five separate ISS body region scores, all regions were significant predictors (Table 3, AIS model). In a subgroup of subjects presenting with hypoperfusion, extremities and pelvic girdle was the only ISS body region group found to independently contribute significantly to fibrinogen concentration with an unstandardised coefficient of –0.146 (*P* = 0.002).

**Table 3:** Linear regression of factors affecting fibrinogen concentration

	**ISS model**		**AIS model**	
	**β (95% CI)**	***P *** **value**	**β (95% CI)**	***P *** **value**
Base excess (mEq/ml)	0.19	< 0.001	0.20	< 0.001
			(0.14, 0.26)	
	(0.13, 0.25)			
Time from injury (minutes)	–0.14	< 0.001	–0.12	< 0.001
			(–0.18, –0.07)	
	(–0.19, –0.08)			
Age (years)	0.27	< 0.001	0.26	< 0.001
			(0.21, 0.31)	
	(0.22, 0.32)			
Gender (male)	–0.19	0.003	–0.19	0.004
			(–0.31, –0.06)	
	(–0.31, –0.06)			
Injury Severity Score	–0.27	< 0.001		
	(–0.33, –0.21)			
Head and neck			–0.11	< 0.001
			(–0.17, –0.06)	
Face			–0.07	0.018
			(–0.12, –0.01)	
Thorax			–0.07	0.035
			(–0.12, –0.00)	
Abdomen and pelvic contents			–0.09	0.002
			(–0.14, 0.03)	
Extremities and pelvic girdle			–0.17	< 0.001
			(–0.22, –0.11)	

AIS, Abbreviated Injury Scale; β, standardised coefficient; CI, confidence interval; ISS, Injury Severity Score.

## Discussion

Key findings from this study include the wide variability in presentation of fibrinogen concentrations, but critically that 8.2% of the patients presented with hypofibrinogenaemia defined as fibrinogen concentration ≤1.5 g/l on admission. There was a strong association between low fibrinogen values and mortality. In our population, survival decreased dramatically with an OR of 0.08 as fibrinogen concentration fell below 2.29 g/l. Risk factors for low fibrinogen concentration were low BE, increasing time from injury, young age, male gender and increasing injury severity. Among injuries in different body regions, a strong contributor to low fibrinogen concentration was the occurrence of severe injuries to the extremities and pelvic girdle.

Current European guidelines for massive haemorrhage following trauma recommend a threshold for substituting fibrinogen at concentrations below 1.5 to 2.0 g/l [14]. The evidence underpinning this practice is very limited and is largely drawn from elective surgery and postpartum haemorrhage, which may not be extrapolated to the trauma setting. Our study identifies a breakpoint for initial fibrinogen concentration around 2.29 g/l, below which the odds of death within 28 days is reduced by a factor of 0.08 for every unit increase in fibrinogen concentration. GAM analysis in the group of patients with fibrinogen concentrations above the breakpoint indicates that mortality tends to increase with increasing fibrinogen concentration (Figure 1a). The confidence interval in this segment is relatively wide and results should be interpreted with caution. Moreover, this relationship was not found to be significant in the piecewise linear regression model.

The association between fibrinogen concentration and mortality has recently been studied by Rourke and colleagues [23]. In a linear regression model they identified an estimated OR of 0.22 for 28-day mortality, slightly lower than in our population with an OR of 0.46. The OR in the linear regression model in our study was significantly different from the lower segment of the piecewise linear regression model with an OR of 0.08 (Table 2). The discrepancy between the linear and piecewise linear regression models indicates that the negative impact of hypofibrinogenaemia in trauma previously may have been underestimated.

The association between fibrinogen concentrations and mortality in our study is in accordance with the findings of Hess and colleagues [19], who demonstrated an increased mortality in subjects with hypofibrinogenaemia, with a comparable curved association between fibrinogen concentration and survival. A possible explanation for the increased mortality in the high fibrinogen concentration group may be the development of thrombotic complications during the post-resuscitation phase [24]. A causative relationship between high fibrinogen levels and post-traumatic thrombotic events, however, is not well established. In fact, a prophylactic infusion of 2 g fibrinogen was shown not to trigger postoperative clinically detectable thromboembolic events in patients undergoing cardiac surgery in a randomised study by Karlsson and colleagues [25]. Despite a transient increase in fibrinogen concentration after infusion and a subsequent reduced blood loss, haemostatic parameters did not differ significantly between groups after 24 hours.

An alternative explanation for the apparent increased mortality in the high fibrinogen group may be related to the fact that fibrinogen is an acute phase protein indicating pre-existing inflammatory disease [26]. High fibrinogen levels are associated with, for example, atherosclerotic disease, which may have been a confounding factor in our analyses [27].

The observation that ISS, age and BE are predictors of mortality in trauma is previously well documented [18,28]. Applying piecewise linear regression, we found a positive linear association between the ISS score and mortality for ISS scores <26. Surprisingly, a slight negative association was found for ISS scores above this breakpoint. This result should, however, be interpreted with caution. A few survivors with very high ISS scores might have biased the effect estimate of this upper segment. A selection bias due to death before reaching inclusion in the study may also have affected the results in the group of most severely injured patients.

Increased INR is also previously documented to be related to mortality [17]. This association was not found to be significant in our study when adjusting for fibrinogen concentration in a piecewise linear model. Platelet count was not found to be an independent predictor of survival when adjusting for fibrinogen concentration in either model. This finding is in accordance with previous studies showing that the apparent relationship between platelet count and mortality becomes insignificant when other coagulation parameters are adjusted for [29,30]. Interestingly, increased survival is reported using a high platelet to red blood cell transfusion ratio compared with lower ratios in trauma patients [31]. This may indicate that not only platelet count, but also platelet function must be taken into consideration [32].

Our analysis of factors contributing to low fibrinogen concentrations demonstrated an association between time from injury and fibrinogen concentration on arrival, even when adjusting for measures of hypoperfusion. One explanation for this association may be the lengthened time frame for the increased consumptive process of fibrinogen previously described in animal studies, regardless of hypothermia and acidosis [6,33].

Young age and male gender were also associated with lower fibrinogen levels in our study population. The fibrinogen concentration in healthy individuals increases with age in both men and women. Females of reproductive age tend to have higher fibrinogen levels than men of the same age, whereas men over the age of 60 have higher levels than women of the same age [34]. The majority of the subjects in our study was below the age of 40, and thus a negative association between male gender and fibrinogen concentration is expected.

Our data identified injury to the extremities and pelvic girdle among the body regions as a strong contributor to hypofibrinogenaemia, such that in the subgroup of hypoperfused patients this was the only type of injury that uniquely contributed to low fibrinogen levels. The impact on coagulation of major orthopaedic trauma including procedures involving intramedullary reaming and nailing has been described [35,36] and may be related to intravasation of procoagulant medullary fat and bone marrow. Increased levels of thrombin–antithrombin complexes on admission after orthopaedic injury have been demonstrated, paralleled by increased levels of tissue plasminogen activator, as a marker of increased fibrinolysis [35]. Fat emboli from the site of injury may also activate coagulation and fibrinolysis in the pulmonary capillaries [37-39].

Our study has some limitations in addition to its partly retrospective nature. Inclusion criteria were not strictly defined, and minor differences between centres may have occurred. Characteristics of the included cohort, however, are reflected in the ISS and clinical markers of injury (Table 1). The body core temperature on admission was missing in a large number of the patients, and was therefore not included in the analyses. Hypothermia is known to reduce fibrinogen synthesis, and the lack of data might have influenced our regression models. On the other hand, the normal acute phase response of fibrinogen synthesis is only evident hours and days after exposure [36]. Within the relatively short time frames in this study, the impact of reduced fibrinogen synthesis is less significant. We did not include data on prehospital fluid administration in this study. The dilutional effect of administered fluids may have reduced the fibrinogen concentration throughout the prehospital phase. A restrictive prehospital fluid regimen is applied in all centres, however, and the potential bias of fluid administration is therefore limited. All centres used the Clauss method to measure fibrinogen concentration. Administration of colloid expanders may overestimate fibrinogen concentration measured by this method [40]. Colloids are extremely rarely used in the prehospital setting in all the four centres, however, and are unlikely to have imposed a systematic bias.

## Conclusions

Our study demonstrates a significant proportion of trauma patients with low fibrinogen levels on arrival in hospital. Fibrinogen concentration on arrival below an estimated threshold value of 2.29 g/l is strongly related to poor outcome. Injuries to the extremities and pelvic girdle, a low BE, long prehospital times, young age and male gender appear to be associated with lower fibrinogen concentrations. These factors should be considered in the early management of exsanguinating patients. The role of fibrinogen supplementation, and identification of subgroups of patients that may benefit from this treatment strategy, should be addressed in new interventional trails.

## Key messages

•Low concentrations of fibrinogen on admission are frequently observed in trauma patients.

•There is a nonlinear relationship between fibrinogen concentrations and 28-day mortality, with a dramatic increase in mortality below a fibrinogen concentration breakpoint value of 2.29 g/l (95% confidence interval: 1.93,2.64).

•Low age, low BE, male gender, high ISS and lengthened time from injury are unique predictors for low fibrinogen concentrations.

•Interventional trials on fibrinogen substitution are recommended.

## Abbreviations

AIS: Abbreviated Injury Scale; BE: base excess; GAM: generalised additive regression model; INR: International Normalized Ratio; ISS: Injury Severity Score; OR: odds ratio.

## Competing interests

The authors declare that they have no competing interests.

## Authors’ contributions

JSH, SS, NPJ, KB, PIJ, PAN and CG made substantial contributions to conception and design of the study. JSH, SS, KB, MJC and TE contributed to acquisition of data. JR and JSH performed the statistical analyses. All authors contributed to interpretation of the data. All authors contributed to revising the manuscript for important intellectual content and have given final approval for the version to be published.
